# Clinical characteristics and disease burden of respiratory syncytial virus infection among hospitalized adults

**DOI:** 10.1038/s41598-020-69017-8

**Published:** 2020-07-21

**Authors:** Jin Gu Yoon, Ji Yun Noh, Won Suk Choi, Jin Ju Park, Yoo Bin Suh, Joon Young Song, Hee Jin Cheong, Woo Joo Kim

**Affiliations:** 1 Division of Infectious Diseases, Department of Internal Medicine, Guro Hospital, Korea University College of Medicine, 148 Gurodong-ro, Guro-gu, Seoul, 08308 South Korea; 2 Division of Infectious Diseases, Department of Internal Medicine, Kangnam Sacred Heart Hospital, Hallym University School of Medicine, Seoul, South Korea; 3 Division of Infectious Diseases, Department of Internal Medicine, Ansan Hospital, Korea University College of Medicine, 123 Cheok-geum-ro, Ansan, Gyeonggi-do, South Korea

**Keywords:** Viral infection, Outcomes research

## Abstract

The disease burden of respiratory syncytial virus (RSV) infection in the adult population has not been well characterized compared to children. Investigation of the clinical characteristics and disease burden of adult RSV infection would help to establish public health policy and a future vaccine strategy. We retrospectively collected medical records of hospitalized adult patients who were diagnosed with RSV infection from January 2012 to December 2015 from three tertiary hospitals. Baseline characteristics, clinical outcomes and economic charge during hospitalization were compared by age groups (19–49 years, 50–64 years, and ≥ 65 years) using Chi-square test. The odds of risk factors of RSV pneumonia were calculated using binary logistic regression. A total of 204 patients from three hospitals were enrolled. Patients who older than 65 years were 132 (64.7%). 118 (57.8%) patients had clinically confirmed pneumonia and 22 (10.8%) died in a hospital. The median medical cost of RSV pneumonia was 2,855.26 USD (interquartile range, 1,561.85–5,379.55) per each admission. Solid cancer (adjusted OR, 3.85; 95% CI, 1.65–9.02, p = 0.002) and hematologic malignancy (all patients had pneumonia) were shown to be risk factors for RSV pneumonia. RSV infection in South Korea seemed to have a significant burden among adults as pneumonia, care in the intensive care unit and mortality. Nationwide awareness and further effort to recognize the current burden, prepare specific treatment, and prevent adult RSV infection would be necessary.

## Introduction

Respiratory syncytial virus (RSV) belongs to the family of *Paramyxoviridae* and is the most important cause of lower respiratory tract infection in infants and young children. The virus causes common cold, bronchiolitis and pneumonia which are spread by close contact and large droplets. However, the clinical impact of RSV infection in adult populations had been underestimated until outbreaks in hospitals and healthcare facilities were identified. The infection may induce serious outcome especially in the elderly, patients with chronic cardiopulmonary disease and immunocompromised individuals^1^. Also, the disease burden of RSV is not negligible compared to influenza among adults at high risk or the elderly^2,3^. In the United States, annually 2–10% of community-dwelling older adults are infected and approximately 11,000 persons die due to the illness^4^. In South Korea, RSV was detected in 1.1% of all adult respiratory infections and shows similar seasonal occurrence during childhood^5,6^.

Recently, the Strategic Advisory Group of Experts of the World Health Organization presented an issue of RSV vaccine production in April 2016, and stated that the elderly, pregnant women and immunocompromised patients would be the target populations of the RSV vaccine. Therefore, a precise investigation of the epidemiology and disease burden of adult RSV infection is key to establishing an accurate vaccine strategy. Furthermore, the increasing elderly population with debilitated host immune response and underlying comorbidities is nowadays a public health problem in developed countries. Unfortunately, large-scale intensive research regarding adult RSV infection is lacking in South Korea. The aim of this study was to reveal the clinical manifestations, epidemiology and disease burden of adult RSV infections in South Korea, especially in elderly and at-risk groups. It may be useful to contemplate the eligible target population of RSV vaccination.

## Methods

### Study subjects and data collection

From January 2012 to December 2015, all adult patients over 19-years-old diagnosed to RSV infection were enrolled retrospectively from three tertiary hospitals: Korea University Guro Hospital, Korea University Ansan Hospital, and Hallym University Kangnam Sacred Heart Hospital. Each hospital has an emergency room, inpatient facility and intensive care unit (ICU) and is located in Seoul and its suburban area.

Participants included all hospitalized RSV-diagnosed patients. The diagnosis of RSV infection was confirmed by viral culture or respiratory viruses multiplex real-time polymerase chain reaction (PCR) kit using respiratory specimens. The viral culture method in the laboratory could not distinguish the serotype of RSV in contrast to real-time PCR kit. We retrospectively reviewed the medical charts of the patients using case report forms, and extracted data regarding epidemiology, duration of admission, ICU admission rate, symptoms, laboratory results, underlying diseases, complications, and clinical outcomes such as pneumonia and use of a mechanical ventilator. Pneumonia was defined as concomitant clinical symptoms, elevation of inflammatory markers and pneumonic infiltration on chest radiograph as confirmed by a radiologist^7^. The ages of the patients were categorized into young adult (19–49 years old), middle-aged adult (50–64 years old), and elderly (65 years old or more).

Medical cost was calculated as all direct healthcare costs including the charge for room and meals, the cost of medicines and expendables, and the cost of any medical procedures^8,9^. The duration of cost summation was from the date of performance of diagnostic test to the date when the patient was transferred to another hospital, discharged or died^10^. Each patient’s cost was extracted in Korean Won (KRW) and presented as U.S. dollars (USD) reflecting the exchange rate at the time (1 USD = 1,128.30 KRW, on 16th October 2018).

This study was approved by the Korea University Institutional Review Board (IRB No. 2019GR0127 in Guro hospital, IRB No. AS16203 in Ansan hospital) and the Kangnam Sacred Heart Hospital Institutional Review Board (IRB No. HKS2019-09-017). The study was performed in accordance with the ethical standards of the Declaration of Helsinki. Informed consent was waived because of the retrospective design of the study.

### Statistical analysis

Each nonparametric variable in baseline characteristics and clinical outcomes was compared using the chi-square test with Fisher’s exact test. Continuous variable such as length of hospital stay was compared using the analysis of variance. The odds of risk factors of RSV pneumonia were calculated using binary logistic regression analysis. Any continuous independent variables were assumed to be a linear relationship with a dependent variable in the logistic regression analysis. A P value of < 0.05 was considered to indicate statistical significance. All statistical analyses were performed using SPSS Statistics version 20 for Windows (IBM Corp., Armonk, NY, USA).

### Ethical approval

2019GR0127 approved by Korea University Institutional Review Board at April 11th, 2019.

## Results

A total of 204 patients were enrolled from the three hospitals: 146 from Guro Hospital, 44 from Ansan Hospital, and 14 from Kangnam Sacred Heart Hospital. Most patients underwent diagnostic tests for RSV because of their newly developed respiratory symptoms and/or lung infiltrations on chest radiograph, except four patients. These four participants were in chronic bedridden states and were incidentally diagnosed with RSV infection during work-up for altered states of consciousness. The demographic characteristics of the patients are shown in Table 1. Sore throat and nasal congestion/rhinorrhea was more dominant in 19–49 years-old group (sore throat: 5 (25.0%); nasal congestion/rhinorrhea: 7 (35.0%)) than in 50–64 years-old group (sore throat: 4 (7.7%); nasal congestion/rhinorrhea: 14 (26.9%)) and ≥ 65 years-old group (sore throat: 6 (4.5%); nasal congestion/rhinorrhea: 21 (15.9%)). All proportions of underlying diseases were not significantly different between age groups. There was no human immunodeficiency virus infection in the study population.

**Table 1:** Baseline characteristics and clinical outcomes of RSV infected patients from 2012 to 2015 distributed by age groups.

Age group	19–49 (n = 20)	50–64 (n = 52)	65– (n = 132)	P value
**Sex (%)**				
Male	10 (50.0)	31 (59.6)	54 (40.9)	0.069
**RSV serotype (%)**				
A	13 (65.0)	36 (69.2)	91 (68.9)	0.510
B	7 (35.0)	11 (21.2)	29 (22.0)	
Untyped	0	5 (9.6)	12 (9.1)	
**Region (%)**				
Seoul	12 (60.0)	22 (42.3)	62 (47.0)	0.624
Incheon and Gyeonggi	7 (35.0)	26 (50.0)	56 (42.4)	
Others	1 (5.0)	4 (7.7)	14 (10.6)	
**Symptoms (%)**				
Any	19 (95.0)	51 (98.1)	130 (98.5)	0.578
Fever	17 (85.0)	31 (59.6)	87 (65.9)	0.124
Cough	13 (65.0)	33 (63.5)	86 (65.2)	0.977
Sputum	11 (55.0)	34 (65.4)	91 (68.9)	0.456
Sore throat	5 (25.0)	4 (7.7)	6 (4.5)	0.005
Nasal congestion/rhinorrhea	7 (35.0)	14 (26.9)	21 (15.9)	0.061
Dyspnea	9 (45.0)	17 (32.7)	55 (41.7)	0.469
**Underlying diseases (%)**				
Any	17 (85.0)	47 (90.4)	120 (90.9)	0.709
Diabetes	6 (30.0)	17 (32.7)	39 (29.5)	0.916
Cardiovascular disease	4 (20.0)	15 (28.8)	36 (27.3)	0.744
Stroke^a^	6 (30.0)	10 (19.2)	26 (19.7)	0.547
Respiratory disease^b^	5 (25.0)	14 (26.9)	38 (28.8)	0.923
Chronic kidney disease	3 (15.0)	5 (9.6)	24 (18.2)	0.354
Liver disease^c^	0	5 (9.6)	10 (7.6)	0.370
Solid cancer	5 (25.0)	13 (25.0)	28 (21.2)	0.826
Hematologic malignancy	3 (15.0)	3 (5.8)	6 (4.5)	0.180
**Clinical data (%)**				
Pneumonia	12 (60.0)	31 (59.6)	75 (56.8)	0.922
ICU care	2 (10.0)	6 (11.5)	33 (25.0)	0.060
Need for mechanical ventilation	0	3 (5.8)	15 (11.4)	0.166
In-hospital mortality	2 (10.0)	6 (11.5)	14 (10.6)	0.976

*SD* standard deviation, *ICU* intensive care unit.

^a^Stroke includes both hemorrhagic and ischemic strokes.

^b^Respiratory disease includes asthma, chronic obstructive pulmonary disease, bronchiectasis and interstitial lung disease.

^c^Liver disease includes chronic hepatitis B and/or C, liver cirrhosis and autoimmune hepatitis.

The general admission rate and proportions of mechanical ventilation, pneumonia and mortality were not significantly different between age groups. There were 12 (60.0%) cases of RSV pneumonia were in the young adult group, 31 (59.6%) in the middle-aged adult group, and 75 (56.8%) in the elderly group. All patients with hematologic malignancies had RSV pneumonia. The rate of ICU admission was higher among the elderly (25.0%) than among young adults (10.0%) and middle-aged adults (11.5%), but also showed no significant difference (p = 0.060) (Table 1).

**Figure 1:**
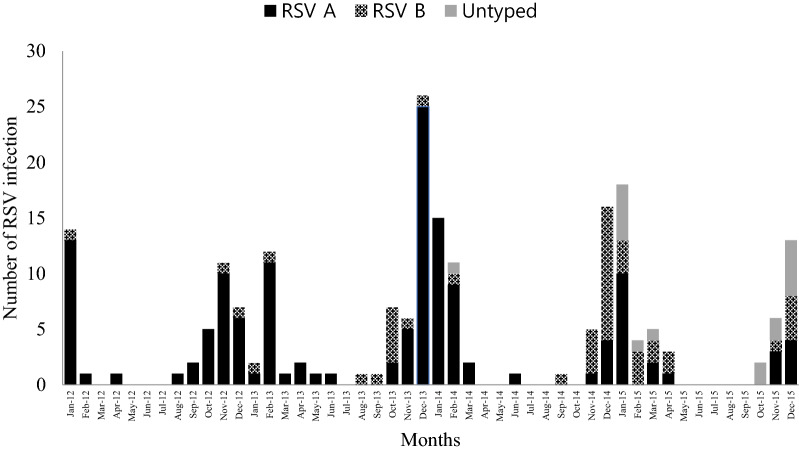
Seasonal distribution of RSV A and B infection from 2012 to 2015.

**Figure 2:**
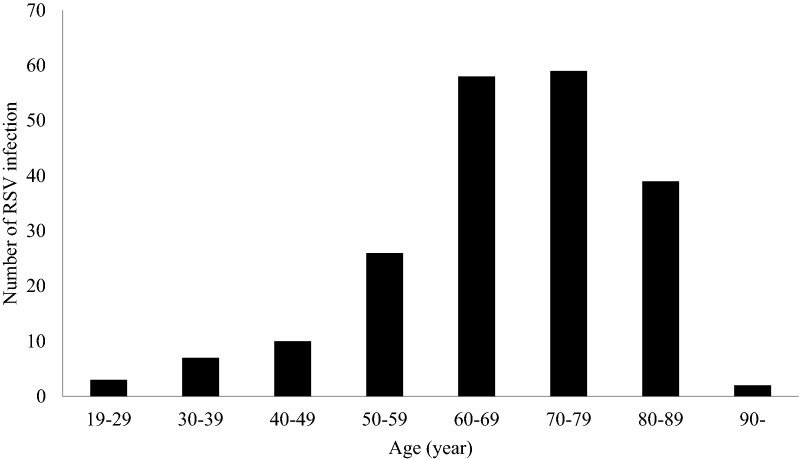
Age distribution of RSV infected patients from 2012 to 2015.

189 (92.6%) were diagnosed by multiplex real-time PCR and 15 (7.4%) were diagnosed by viral culture. The results of RSV serotype in 17 patients were undefined, including two missed data in PCR group. The dominant serotype of RSV changed annually and prevailed from winter to early spring. RSV serotype A was dominant in 2012–2013 and 2013–2014 winter seasons (Fig. 1). More than half of patients enrolled in the study were in the elderly group (65 years old or more, 64.7%) and the mean age ± standard deviation was 67.77 ± 13.94 (Fig. 2).

The median medical cost of RSV pneumonia per each admission seemed to be increasing by the age groups: 1,957.33 USD in the young adult group, 2,116.43 USD in the middle-aged group, and 2,933.17 USD in the elderly group. The differences in mean medical cost were not statistically significant between age groups and underlying diseases (data not shown). Admission rate, mortality and length of hospital stay also showed no statistical difference between age groups (Table 2).

**Table 2:** Clinical outcomes and medical cost of RSV pneumonia distributed by age groups.

Age	19–49 (n = 12)	50–64 (n = 31)	65– (n = 75)	Total (N = 118)	P value
**Outcome (%)**					
ICU care	1 (8.3)	5 (16.1)	20 (26.7)	26 (22.0)	0.237
Need for mechanical ventilation	0	3 (9.7)	11 (14.7)	14 (11.9)	0.313
In-hospital mortality	2 (16.7)	5 (16.1)	9 (12.0)	16 (13.6)	0.807
Length of hospital stay (days) (mean ± SD)	19.17 ± 26.66	17.00 ± 27.31	20.40 ± 33.63	19.38 ± 31.23	0.880
**Economic burden**					
Medical cost (USD) per each admission, [median (IQR)]	1,957.33 (1,332.49–10,117.20)	2,116.43 (1,520.64–4,232.15)	2,933.17 (1,748.26–6,339.93)	2,855.26 (1,561.85–5,379.55)	

*USD* US dollar, *IQR* interquartile range, *ICU* intensive care unit, *SD* standard deviation.

To evaluate which risk factors contribute to the occurrence of pneumonia in RSV infection, odds ratios (ORs) were calculated for each age group, sex, symptoms, RSV serotype and underlying disease (Table 3). Age group, sex, RSV serotype and symptoms were not related to RSV pneumonia. Regarding underlying diseases, solid cancer (adjusted OR, 3.85; 95% CI, 1.65–9.02, p = 0.002) and hematologic malignancy (all patients had pneumonia) appeared to be significantly associated with RSV pneumonia.

**Table 3:** Predicted risk factors of RSV pneumonia calculated by binary logistic regression analysis.

	Proportion of RSV pneumonia (%)	Odds ratio (95% CI)			
		Crude	P value	Adjusted*	P value
**Sex**					
Female	59/109 (54.1)	Reference		Reference	
Male	59/95 (62.1)	1.39 (0.79–2.43)	0.250	1.47 (0.80–2.71)	0.215
**Age group**					
19–49 years	12/20 (60.0)	Reference		Reference	
50–64 years	31/52 (59.6)	0.98 (0.34–2.82)	0.976	0.68 (0.22–2.12)	0.511
≥ 65 years	75/132 (56.8)	0.88 (0.34–2.29)	0.789	0.70 (0.25–1.94)	0.489
**RSV serotype**					
A	84/140 (60.0)	1.71 (0.88–3.32)	0.116	1.53 (0.75–3.12)	0.244
B	22/47 (46.8)	Reference		Reference	
Symptoms**					
Not exist	1/4 (25.0)	Reference		Reference	
Exist	117/200 (58.5)	4.23 (0.43–41.37)	0.205	6.72 (0.57–78.84)	0.129
**Underlying diseases**					
Diabetes	35/62 (56.5)	0.92 (0.50–1.68)	0.790	1.00 (0.51–1.95)	0.999
Cardiovascular disease	32/55 (58.2)	1.02 (0.55–1.91)	0.953	1.38 (0.69–2.77)	0.367
Stroke	20/42 (47.6)	0.59 (0.30–1.18)	0.134	0.61 (0.29–1.28)	0.187
Respiratory disease	35/57 (61.4)	1.23 (0.66–2.29)	0.522	1.25 (0.64–2.44)	0.512
Chronic kidney disease	16/32 (50.0)	0.69 (0.32–1.46)	0.330	0.89 (0.40–1.99)	0.887
Solid cancer	36/46 (78.3)	3.34 (1.55–7.18)	0.002	3.85 (1.65–9.02)	0.002
Hematologic malignancy	12/12 (100)	+ INF		–	–

*+ INF* plus infinity.

*Adjusted for age, sex, RSV serotype, symptoms, and underlying disease except for hematologic malignancy.

**Symptoms include fever, cough, sputum, sore throat, nasal congestion/rhinorrhea and dyspnea.

## Discussion

Our findings suggested that considerable mortality and economic burden of RSV infection in the adult population exist in South Korea. In addition, solid cancer and hematologic malignancy displayed significant relationships with RSV pneumonia.

Recently, the importance of RSV infection in the adult population is increasing globally. However, there have been insufficient studies about the clinical characteristics and disease burden of adult RSV infection. In contrast, studies of RSV infection in childhood are well known and actively conducted in various countries and groups. Hall et al. showed that 18% of children with acute respiratory infections under the age of 5 years had RSV infection. This high infection rate is a serious problem in pediatric public health because it leads to a substantial disease burden regardless of individual underlying illnesses^11,12^. On the other hand, RSV infection in adults seems to be associated with the elderly population and chronic diseases despite the lower rate than in childhood. In a study conducted in the U.S., RSV infection comprised 8.3% of acute respiratory illnesses among the elderly; this proportion was 4% in a French study^13,14^. In Korean studies, laboratory-confirmed RSV infection accounted for 2.8% of all respiratory viral infections and 12.7% of hospitalizations on account of respiratory viral infection^15,16^. However, the percentage of RSV among all respiratory illnesses including bacterial infection in Korean adults had not been reported.

The seasonal trend of RSV serotype predominance seemed to be alternated biannually, which was also shown in other countries^17^. This pattern might be explained by the acquisition of transient herd immunity of average duration 2 years^18^. It is controversial in children and infants that the serotype variability has an impact on clinical course and outcome of RSV infection^19^. However, none of studies in our knowledge presented precise difference of clinical manifestations between RSV serotype A and B in adults. In our study, the RSV serotype did not seem to be a risk factor of RSV pneumonia. Further clinical and physiologic research would be necessary to clarify the effect of RSV serotype to clinical outcomes.

Most RSV patients were over 65 years of age. Nonhospitalized RSV infected patients were excluded in our study, but they might impose a considerable burden on local clinics as influenza-like illnesses. Nevertheless, the high rates of ICU admission (25.0%), pneumonia (56.8%) and in-hospital mortality (10.6%) in the elderly population signify the severity of adult RSV infection. Lee et al. also reported that 71.9% of adults with RSV infection had lower respiratory complications, and 9.1% died within 30 days^7^. The rate of mortality in our study was elevated from 10.8 to 13.6% when the population was confined to that with pneumonia, which seems to be important in determining the prognosis of RSV infections.

The economic burden of adult RSV infection is not well known in contrast to that among children. In the U.S., RSV infection had similar burden and clinical outcomes to influenza infection in the adult population^2^. Han et al. calculated almost 20 years ago that the mean cost of RSV pneumonia during hospitalization was $11,000^20^. The relatively low cost in our study may be attributed to the discrepancy of medical charge and national insurance systems between the two countries. We set the median medical cost to amend the skewed distribution of individual medical costs. The median medical cost was similar between age groups which may reflect a high prevalence of underlying diseases in all groups. However, a larger and more intensive study of the economic burden of adult RSV infection would be necessary to clarify this pattern. The entire domestic cost of adult RSV infection would be much more increased because of easy diagnosis, aging of the population, and an increasing prevalence of concomitant chronic diseases.

The presence of underlying disease affected the poor outcome of RSV infection in previous studies, especially cancerous or immunocompromised status. Solid organ transplantation and hematopoietic stem cell transplantation were related to increased burden of RSV infection in many studies, and the outcomes worsened when the patients had lower respiratory diseases^21,22^. A study of South Korean adults also showed that chronic lung disease, bacterial co-infection and lower respiratory infection could be associated with increased mortality^23^. Our data revealed that solid cancer and hematologic malignancy were significantly related to the occurrence of RSV pneumonia; however, other underlying illnesses, RSV serotype, sex, and age were not related. This suggests that even young adults could suffer severe RSV infection and bear considerable disease burden.

The study has several limitations. First, we could not obtain the general incidence of RSV infection in the entire population due to the retrospective study design and the limited area of residence among the participants. In addition, the unbalanced sampling from each participating hospital might lead to selection bias because most of cases were collected from one hospital [Guro hospital: 146 (70.2%)]. Differences of baseline characteristics between three hospitals were shown in Supplementary Table 1. Second, RSV infections with mild symptoms would be overlooked by low rate of PCR performance, clinicians’ unawareness and lack of active antiviral agents^24^. This ignorance might underestimate the burden of RSV infection. Third, cases of some underlying diseases and other variables like liver disease and HIV infection were so few that related data might be unreliable. Nevertheless, our study would be helpful to recognize the magnitude of RSV infection in the hospital and to facilitate to make an appropriate countermeasure against adult RSV infection through vaccine development, novel antiviral agents and control of nosocomial transmission.

In conclusion, RSV infection in the adult population showed significant clinical and economic burden and largely related to lower respiratory tract complications and mortality. Solid cancer and hematologic malignancies contributed to the occurrence of RSV pneumonia. The substantial burden of adult RSV infection should be considered to establish public health policies and the target of the future RSV vaccine.

## Supplementary information

Supplementary Table 1.
