# Supplementary material for: Clinical characteristics and disease burden of respiratory syncytial virus infection among hospitalized adults
Source: Sci Rep. 2020 Jul 21;10:12106. doi: 10.1038/s41598-020-69017-8 (PMC7374583; doi:10.1038/s41598-020-69017-8)
Supplement: Supplementary file 1 — Supplementary Table 1. [file 41598_2020_69017_MOESM1_ESM.docx]

**Clinical Characteristics and Disease Burden of Respiratory Syncytial Virus Infection**

**Among Hospitalized Adults**

Jin Gu Yoon^1^, Ji Yun Noh^1^, Won Suk Choi^1^, Jin Ju Park^2^, Yoo Bin Suh^2^, Joon Young Song^1^, Hee Jin Cheong^1*^, and Woo Joo Kim^1^

**Affiliations**

1. Division of Infectious Diseases, Department of Internal Medicine, Korea University College of Medicine, Seoul, Korea

2. Division of Infectious Diseases, Department of Internal Medicine, Kangnam Sacred Heart Hospital, Hallym University School of Medicine, Seoul, Korea

**Keywords**: Respiratory syncytial viruses, Pneumonia, Risk factors, Cost of illness

**Contributions:** JG Yoon, JY Noh, JY Song and HJ Cheong designed the study. WS Choi, JJ Park and YB Suh supported data collection and analysis. JG Yoon also analyzed data and wrote the paper. All authors contributed to the discussion, edited the paper and confirmed the final version.

**Correspondence:** Hee Jin Cheong, MD, PhD. Division of Infectious Diseases, Department of Internal Medicine, Guro Hospital, Korea University College of Medicine, 148 Gurodong-ro, Guro-gu, Seoul, (08308) Korea. Tel: 82-2-2626-3050, Fax: 82-2-2626-1105, E-mail: heejinmd@korea.ac.kr

**Supplementary table 1. Baseline characteristics and clinical outcomes of RSV infected patients from 2012 to 2015 distributed by participating hospitals**

| **Age group** | **Guro hospital**  **(n=146)** | **Ansan hospital**  **(n=44)** | **Kangnam Sacred Heart hospital**  **(n=14)** | **P value** |
| --- | --- | --- | --- | --- |
| **Sex (%)** |  |  |  |  |
| Male | 73 (50.0) | 18 (40.9) | 4 (28.6) | .214 |
| **RSV serotype (%)** |  |  |  |  |
| A | 116 (79.5) | 19 (43.2) | 5 (35.7) | <.001 |
| B | 30 (20.5) | 9 (20.5) | 8 (57.1) |  |
| Untyped | 0 | 16 (36.4) | 1 (7.1) |  |
| **Age group** |  |  |  |  |
| 19-49 years | 17 (11.6) | 2 (4.5) | 1 (7.1) | .580 |
| 50-64 years | 35 (24.0) | 14 (31.8) | 3 (21.4) |  |
| ≥65 years | 94 (64.4) | 28 (63.6) | 10 (71.4) |  |
| **Region (%)** |  |  |  |  |
| Seoul | 85 (58.2) | 1 (2.3) | 10 (71.4) | <.001 |
| Incheon and Gyeonggi | 45 (30.8) | 40 (90.9) | 4 (28.6) |  |
| Others | 16 (11.0) | 3 (6.8) | 0 |  |
| **Symptoms (%)** |  |  |  |  |
| Any | 142 (97.3) | 44 (100) | 14 (100) | .445 |
| Fever | 97 (66.4) | 31 (70.5) | 7 (50.0) | .368 |
| Cough | 101 (69.2) | 20 (45.5) | 11 (78.6) | .008 |
| Sputum | 102 (69.9) | 24 (54.5) | 10 (71.4) | .155 |
| Sore throat | 5 (3.4) | 7 (15.9) | 3 (21.4) | .002 |
| Nasal congestion  /Rhinorrhea | 37 (25.3) | 1 (2.3) | 4 (28.6) | .003 |
| Dyspnea | 57 (39.0) | 20 (45.5) | 4 (28.6) | .507 |
| **Underlying diseases (%)** |  |  |  |  |
| Any | 132 (90.4) | 39 (88.6) | 13 (92.9) | .887 |
| Diabetes | 40 (27.4) | 17 (38.6) | 5 (35.7) | .330 |
| Cardiovascular disease | 40 (27.4) | 13 (29.5) | 2 (14.3) | .521 |
| Stroke^a^ | 29 (19.9) | 11 (25.0) | 2 (14.3) | .634 |
| Respiratory disease^b^ | 36 (24.7) | 15 (34.1) | 6 (42.9) | .206 |
| Chronic kidney disease | 16 (11.0) | 14 (31.8) | 2 (14.3) | .004 |
| Liver disease^c^ | 8 (5.5) | 5 (11.4) | 2 (14.3) | .249 |
| Solid cancer | 46 (31.5) | 0 | 0 | <.001 |
| Hematologic malignancy | 12 (8.2) | 0 | 0 | .079 |
| **Clinical data (%)** |  |  |  |  |
| Pneumonia | 88 (60.3) | 26 (59.1) | 4 (28.6) | .071 |
| ICU care | 30 (20.5) | 7 (15.9) | 4 (28.6) | .570 |
| Need for mechanical ventilation | 13 (8.9) | 5 (11.4) | 0 | .426 |
| In-hospital mortality | 19 (13.0) | 1 (2.3) | 2 (14.3) | .120 |

^a^ Stroke includes both hemorrhagic and ischemic strokes

^b^ Respiratory disease includes asthma, chronic obstructive pulmonary disease, bronchiectasis and interstitial lung disease

^c^ Liver disease includes chronic hepatitis B and/or C, liver cirrhosis and autoimmune hepatitis

SD, standard deviation; ICU, intensive care unit
